# Supplementary material for: A Likelihood Approach for Real-Time Calibration of Stochastic Compartmental Epidemic Models
Source: PLoS Comput Biol. 2017 Jan 17;13(1):e1005257. doi: 10.1371/journal.pcbi.1005257 (PMC5240920; doi:10.1371/journal.pcbi.1005257)
Supplement: S1 File — (TAR.GZ) [file pcbi.1005257.s014.tar.gz › HSPH_Online-SI-Revision/Additions/MSS11/n100-extreme-SEITR/n100-extreme-SEITR_NEW_table-8.pdf]

1	R0	{1.38654, 9.85565, 0.1}	{2.33206, 13.9544, 0.}	{{1.47608, 4.06661, 0.}, {2.48143, 15.7026, 0.}}	{68.1924, 57.0651, 100.}	inf	521.	658.626	{275.372, 768.471}	40.6568	0.167574	newly	522.	481.81	{400., 564.}	15.6954	0.0703556	Reff	1.36736	2.30457	{1.46547, 2.45314}	68.5409	ar	92379.	84396.5	{56832., 89302.}	8.67916
2	R0	{2.36629, 12.2029, 0.1}	{2.01596, 14.0458, 0.44}	{{1.38815, 7.29751, 0.}, {2.73108, 19.1609, 1.}}	{19.6677, 31.0605, 476.}	inf	14.	6.42591	{1.95697, 12.4042}	56.6484	0.442354	newly	20.	5.11	{1., 10.}	77.95	0.860392	Reff	2.3656	2.01572	{1.38795, 2.73045}	19.6599	ar	99022.	64244.4	{10., 91847.}	35.1211
3	R0	{2.32253, 12.1858, 1.}	{2.08856, 7.50964, 0.49}	{{1.24751, 2.36445, 0.}, {2.8186, 13.6009, 2.}}	{21.6896, 47.8015, 79.}	inf	18.	7.07555	{1.96037, 13.3859}	73.8049	0.678365	newly	26.	20.94	{2., 42.}	79.7692	0.502912	Reff	2.32139	2.08832	{1.24741, 2.8183}	21.686	ar	98997.	69678.8	{64., 92600.}	29.6152
4	R0	{1.79075, 11.2058, 1.}	{1.3832, 3.62731, 0.03}	{{1.22544, 2.5364, 0.}, {1.53501, 4.06661, 0.}}	{23.6281, 67.6301, 99.}	inf	68.	30.0783	{7.0812, 55.3731}	68.9914	0.624248	newly	88.	86.91	{19., 168.}	51.9432	0.28908	Reff	1.78676	1.38197	{1.22418, 1.53374}	23.5339	ar	96920.	44228.2	{13846., 61314.}	54.3663
5	R0	{2.26952, 9.62371, 0.1}	{1.84831, 13.385, 0.42}	{{1.13726, 6.0871, 0.}, {2.7499, 18.9003, 1.}}	{27.5374, 53.1382, 456.}	inf	2.	3.31397	{1.53399, 5.10012}	52.7372	0.307271	newly	5.	10.76	{0., 9.}	226.8	0.645311	Reff	2.26934	1.84823	{1.1372, 2.74978}	27.5363	ar	98881.	50492.6	{4., 91787.}	48.936
6	R0	{1.57191, 6.89004, 0.1}	{2.11714, 11.9618, 0.7}	{{1.25474, 5.71232, 0.}, {2.93371, 18.1089, 2.}}	{43.1774, 82.4481, 704.}	inf	11.	7.75173	{2.62471, 13.6854}	49.6907	0.325856	newly	22.	8.46	{1., 17.}	73.6364	0.683551	Reff	1.57147	2.11685	{1.25459, 2.93317}	43.1894	ar	95046.	69616.6	{28., 93474.}	26.7548
7	R0	{1.99226, 17.2339, 1.}	{2.17051, 12.2958, 0.07}	{{1.44762, 4.00571, 0.}, {2.29055, 17.7413, 0.}}	{18.5682, 29.3419, 101.}	inf	713.	719.543	{302.689, 855.174}	32.5333	0.20856	newly	648.	579.22	{511., 662.}	14.9907	0.0721572	Reff	1.94486	2.1405	{1.43499, 2.25946}	19.3091	ar	97992.	80611.2	{55325., 86383.}	17.7369
8	R0	{2.63369, 15.1879, 0.1}	{2.27921, 11.4957, 1.14}	{{1.53501, 5.43578, 0.}, {2.8833, 17.2484, 2.}}	{17.5365, 31.2407, 1104.}	inf	35.	27.5323	{9.5277, 46.6453}	48.0031	0.343851	newly	41.	32.03	{12., 50.}	52.4146	0.292862	Reff	2.6309	2.27811	{1.53437, 2.88137}	17.5135	ar	99451.	81693.4	{60397., 93278.}	17.8557
9	R0	{2.53271, 9.90552, 0.1}	{1.96894, 14.6631, 0.46}	{{1.16318, 9.219, 0.}, {2.74056, 19.3966, 1.}}	{26.4066, 52.5466, 486.}	inf	2.	3.14548	{1.25269, 5.91079}	77.0784	0.223898	newly	8.	2.37	{0., 5.}	74.125	0.827517	Reff	2.53258	1.96885	{1.16315, 2.7405}	26.4061	ar	99355.	56423.6	{3., 91189.}	43.2101
10	R0	{2.48791, 18.9003, 2.}	{2.42681, 10.9685, 0.54}	{{2.03307, 8.25876, 0.}, {2.73634, 13.5542, 1.}}	{9.58523, 41.9667, 73.}	inf	205.	162.387	{109.398, 216.243}	47.7856	0.299724	newly	173.	172.79	{121., 223.}	19.0809	0.084242	Reff	2.47216	2.41995	{2.02786, 2.72834}	9.57082	ar	99291.	87283.8	{80823., 91879.}	12.0929
11	R0	{2.84414, 18.4765, 1.}	{2.34374, 13.6544, 0.34}	{{1.75359, 8.56054, 0.}, {2.76795, 19.255, 1.}}	{18.0874, 27.3804, 70.}	inf	68.	71.7741	{31.3619, 104.424}	32.4402	0.206701	newly	76.	54.83	{34., 77.}	32.75	0.1892	Reff	2.83774	2.34071	{1.75198, 2.7643}	18.0216	ar	99654.	84386.8	{71343., 92301.}	15.3202
12	R0	{2.29369, 14.622, 0.1}	{2.42298, 12.0771, 0.41}	{{1.93419, 6.25659, 0.}, {2.93371, 17.1903, 1.}}	{15.0789, 24.9822, 438.}	inf	100.	80.3269	{40.5542, 112.795}	43.0306	0.292411	newly	114.	73.55	{46., 99.}	36.4123	0.219748	Reff	2.28683	2.41953	{1.93215, 2.92899}	15.1715	ar	98982.	85233.4	{78023., 93634.}	13.89
13	R0	{2.73413, 16.7529, 1.}	{2.35432, 10.5729, 0.4}	{{1.74092, 5.83416, 0.}, {2.86001, 13.5542, 1.}}	{15.0868, 37.6404, 70.}	inf	62.	49.2573	{26.5693, 69.2025}	37.9084	0.243709	newly	67.	55.46	{29., 88.}	31.0746	0.172896	Reff	2.72932	2.35227	{1.73983, 2.85645}	15.0314	ar	99543.	83758.2	{71160., 92889.}	15.8573
14	R0	{1.79554, 16.5943, 2.}	{2.12656, 13.0929, 0.}	{{2.11292, 12.9719, 0.}, {2.11292, 12.9719, 0.}}	{18.4356, 21.2485, 100.}	inf	652.	738.727	{670.516, 791.591}	23.3496	0.116148	newly	541.	533.41	{478., 585.}	6.14233	0.0271767	Reff	1.75512	2.09709	{2.08265, 2.08528}	19.4838	ar	96821.	82752.2	{82253., 82865.}	14.5308
15	R0	{1.91429, 13.7771, 1.}	{1.6596, 5.25936, 0.12}	{{1.44762, 4.00571, 0.}, {1.74592, 5.83416, 0.}}	{13.3045, 61.8253, 94.}	inf	266.	164.533	{87.0248, 238.044}	56.5875	0.39478	newly	278.	345.65	{210., 471.}	37.3705	0.144219	Reff	1.89691	1.65247	{1.44154, 1.73943}	12.8863	ar	97541.	65992.6	{54435., 71404.}	32.3437
16	R0	{1.42094, 10.5871, 2.}	{2.27954, 12.4005, 1.19}	{{1.24751, 2.5364, 0.}, {2.97157, 19.3547, 2.}}	{65.5924, 69.297, 40.5}	inf	260.	417.854	{71.0687, 680.68}	63.171	0.341112	newly	379.	355.16	{257., 451.}	20.285	0.0942483	Reff	1.40736	2.26099	{1.2423, 2.94363}	65.6098	ar	93044.	75004.5	{37017., 93914.}	20.1299
17	R0	{1.45515, 6.16466, 0.1}	{1.93578, 15.0006, 0.38}	{{1.11927, 9.98575, 0.}, {2.73108, 19.4475, 1.}}	{43.0257, 144.086, 414.}	inf	5.	3.04834	{1.45911, 5.43435}	43.2883	0.185467	newly	11.	2.63	{0., 5.}	83.5455	1.01616	Reff	1.45502	1.9357	{1.11923, 2.73097}	43.0296	ar	93254.	58717.1	{4., 91854.}	37.0973
18	R0	{2.85669, 13.2416, 0.1}	{2.03976, 13.118, 0.73}	{{1.21457, 8.26611, 0.}, {2.93488, 18.4377, 2.}}	{29.5146, 26.068, 720.}	inf	3.	5.6578	{1.81017, 9.64533}	53.1714	0.390091	newly	8.	5.2	{1., 12.}	69.5	0.52982	Reff	2.85618	2.03955	{1.2145, 2.93454}	29.5107	ar	99644.	66278.1	{14., 93508.}	33.4851
19	R0	{2.56417, 17.9558, 0.1}	{2.26405, 13.5325, 0.18}	{{1.53501, 4.06661, 0.}, {2.81891, 18.2812, 1.}}	{15.4845, 25.74, 248.}	inf	91.	90.7789	{35.3187, 126.294}	41.9362	0.288666	newly	91.	68.28	{44., 90.}	30.5934	0.170787	Reff	2.55604	2.26027	{1.53359, 2.81347}	15.3952	ar	99389.	82430.4	{60795., 92690.}	17.0629
20	R0	{2.27517, 11.1864, 0.1}	{2.44013, 9.91956, 0.51}	{{1.74592, 4.40955, 0.}, {2.93906, 14.5291, 2.}}	{18.4876, 27.1872, 534.}	inf	8.	11.9987	{2.87151, 21.1919}	42.5771	0.280187	newly	17.	19.24	{3., 45.}	71.8824	0.338859	Reff	2.27446	2.43965	{1.74568, 2.93829}	18.4914	ar	98894.	81981.9	{67530., 93638.}	17.1012
21	R0	{2.35735, 10.4735, 0.1}	{2.07726, 11.7481, 0.53}	{{1.38427, 6.00596, 0.}, {2.80387, 18.4249, 2.}}	{19.883, 33.1202, 554.}	inf	5.	3.99398	{1.39206, 7.87286}	48.7812	0.276369	newly	5.	4.82	{0., 12.}	78.4	0.496171	Reff	2.35707	2.07713	{1.38421, 2.80374}	19.8812	ar	99036.	63220.8	{5., 92524.}	36.1638
22	R0	{1.79551, 11.9179, 2.}	{2.5184, 10.3957, 0.5}	{{1.9862, 6.28417, 0.}, {2.99939, 13.7076, 1.}}	{40.6121, 20.3635, 75.}	inf	143.	239.838	{149.037, 324.052}	25.8512	0.11669	newly	205.	279.87	{213., 355.}	40.2293	0.143753	Reff	1.78616	2.50823	{1.98151, 2.98335}	40.7673	ar	97803.	88132.9	{79462., 94039.}	9.17223
23	R0	{1.23545, 6.31687, 0.1}	{1.82133, 4.83852, 0.18}	{{1.39162, 1.89883, 0.}, {2.14326, 6.28417, 1.}}	{47.4222, 35.7092, 258.}	inf	66.	96.97	{30.8942, 161.037}	42.1943	0.272313	newly	213.	338.91	{118., 635.}	87.7512	0.268357	Reff	1.23161	1.81724	{1.38876, 2.1375}	47.5506	ar	88739.	70534.1	{50688., 83119.}	20.8807
24	R0	{2.24089, 10.1789, 0.1}	{1.77126, 14.3319, 0.28}	{{1.11927, 8.89248, 0.}, {2.56757, 19.2013, 1.}}	{27.2057, 46.9174, 334.}	inf	4.	3.34194	{1.27522, 6.12516}	54.0654	0.371774	newly	6.	2.3	{0., 5.}	72.3333	0.777438	Reff	2.24062	1.77116	{1.11922, 2.56742}	27.2035	ar	98868.	45565.8	{4., 89105.}	53.9125
25	R0	{1.57298, 10.2607, 0.1}	{2.47721, 12.3414, 0.12}	{{2.15677, 10.1363, 0.}, {2.96947, 15.963, 1.}}	{57.4854, 22.582, 198.}	inf	107.	170.251	{124.812, 209.434}	25.8488	0.0996428	newly	146.	151.09	{114., 187.}	17.4041	0.0739124	Reff	1.56721	2.47002	{2.15145, 2.95851}	57.6063	ar	94957.	88359.8	{83726., 93726.}	6.94756
26	R0	{1.2154, 8.2576, 0.1}	{2.63822, 14.6017, 0.69}	{{2.15677, 10.1363, 0.}, {2.93371, 17.1903, 1.}}	{117.066, 80.2584, 656.}	inf	374.	963.462	{597.177, 1196.04}	110.338	0.314492	newly	559.	687.06	{613., 758.}	23.8784	0.0920648	Reff	1.19834	2.5968	{2.12734, 2.88482}	116.7	ar	88421.	88929.2	{83102., 93720.}	7.11588
27	R0	{2.22841, 18.7983, 2.}	{2.58422, 14.8668, 0.58}	{{2.14747, 10.7247, 0.}, {2.90165, 18.0404, 1.}}	{18.1679, 20.9139, 71.}	inf	579.	634.604	{433.968, 786.977}	25.2015	0.137644	newly	487.	439.42	{394., 481.}	11.2936	0.0527155	Reff	2.1875	2.55685	{2.12943, 2.86805}	18.8213	ar	98756.	89229.2	{83370., 93416.}	9.64685
28	R0	{1.27778, 5.36767, 1.}	{2.23351, 3.29811, 0.11}	{{1.39162, 1.89883, 0.}, {2.29949, 5.42517, 0.}}	{75.2204, 38.7917, 91.}	inf	15.	21.7724	{5.37267, 40.0384}	65.2275	0.286019	newly	37.	282.78	{54., 557.}	668.324	0.707963	Reff	1.27717	2.23265	{1.39128, 2.29734}	75.2357	ar	89843.	77468.	{49770., 93865.}	16.5061
29	R0	{2.38042, 15.8901, 2.}	{2.27341, 9.08766, 0.39}	{{1.737, 4.61852, 0.}, {2.75598, 12.7047, 1.}}	{16.3822, 42.8093, 80.5}	inf	75.	81.2728	{36.4252, 126.081}	38.4714	0.2474	newly	93.	107.56	{68., 154.}	32.9892	0.128309	Reff	2.37421	2.27009	{1.73505, 2.75216}	16.36	ar	99112.	82064.6	{70281., 92259.}	17.2001
30	R0	{2.53188, 12.7607, 0.1}	{1.95283, 13.8718, 0.38}	{{1.12882, 8.35044, 0.}, {2.77394, 18.8857, 1.}}	{26.896, 27.0379, 422.}	inf	1.	2.96203	{1.27686, 5.27281}	48.4759	0.315366	newly	3.	2.64	{0., 6.}	80.6667	0.552686	Reff	2.5317	1.95274	{1.12878, 2.77376}	26.8946	ar	99317.	56050.8	{3., 91965.}	43.5638
31	R0	{1.24482, 10.5471, 1.}	{1.26297, 3.44632, 0.04}	{{1.22544, 3.07157, 0.}, {1.28596, 3.98513, 0.}}	{2.57896, 67.817, 98.}	inf	904.	448.735	{297.982, 556.177}	65.432	0.476092	newly	1087.	1159.83	{843., 1394.}	16.4241	0.0701696	Reff	1.19689	1.23603	{1.19954, 1.25733}	3.2696	ar	89097.	37870.2	{34027., 40831.}	57.4955
32	R0	{2.30446, 17.9607, 1.}	{2.26861, 10.268, 1.11}	{{1.47608, 4.02511, 0.}, {2.76203, 15.0558, 2.}}	{19.1328, 42.8307, 89.}	inf	288.	204.139	{75.5742, 299.541}	52.1922	0.380817	newly	254.	219.82	{162., 286.}	22.0787	0.114767	Reff	2.28427	2.25995	{1.47301, 2.75066}	19.2279	ar	99028.	81375.3	{57024., 92193.}	17.826
33	R0	{2.31579, 11.8236, 2.}	{1.93588, 3.73295, 0.03}	{{1.06738, 2.19254, 0.}, {2.90743, 5.42517, 0.}}	{29.0125, 68.428, 98.5}	inf	22.	13.7445	{2.63314, 27.9653}	56.7163	0.477362	newly	33.	121.85	{7., 194.}	305.667	0.479933	Reff	2.31442	1.9354	{1.06726, 2.90622}	29.0062	ar	99012.	62314.2	{34., 93035.}	37.064
34	R0	{1.6678, 10.6744, 1.}	{2.30649, 9.08127, 0.35}	{{1.53501, 4.02511, 0.}, {2.75598, 12.8326, 1.}}	{42.0016, 29.1376, 65.}	inf	174.	175.93	{75.5514, 275.69}	33.0798	0.208832	newly	267.	230.77	{150., 307.}	22.7903	0.11798	Reff	1.65861	2.29903	{1.53102, 2.7458}	42.2298	ar	95809.	82510.1	{59429., 92248.}	13.8807
35	R0	{1.65476, 7.07559, 0.1}	{2.09357, 11.3971, 0.46}	{{1.14344, 5.58477, 0.}, {2.90743, 18.1089, 1.}}	{38.9681, 70.7179, 480.}	inf	1.	4.38002	{1.40432, 7.56874}	53.1116	0.375799	newly	10.	6.63	{1., 17.}	82.7	0.606047	Reff	1.65458	2.09343	{1.1434, 2.9072}	38.9705	ar	95746.	59645.	{5., 93292.}	37.705
36	R0	{2.71807, 18.5959, 0.1}	{2.51471, 12.9508, 0.22}	{{2.13505, 8.56054, 0.}, {2.96947, 15.963, 1.}}	{12.7105, 30.4103, 276.}	inf	74.	62.8128	{33.7425, 85.8348}	30.1401	0.185957	newly	61.	54.85	{34., 71.}	26.7705	0.130132	Reff	2.71258	2.51209	{2.13316, 2.96621}	12.6882	ar	99540.	87087.7	{82842., 93859.}	12.5098
37	R0	{2.6202, 12.7552, 0.1}	{1.29665, 16.754, 0.33}	{{1.03756, 12.483, 0.}, {1.59945, 19.8898, 1.}}	{50.5135, 35.3836, 368.}	inf	2.	2.93631	{1.18641, 5.72334}	61.0817	0.470562	newly	7.	1.27	{0., 3.}	81.8571	1.01794	Reff	2.61991	1.29658	{1.03751, 1.59938}	50.5106	ar	99462.	21715.5	{4., 64251.}	78.1592
38	R0	{1.47672, 9.81651, 1.}	{2.54273, 12.8851, 0.25}	{{2.32891, 10.9956, 0.}, {2.93371, 17.1903, 1.}}	{72.1879, 31.4483, 75.}	inf	196.	410.281	{329.616, 499.253}	25.135	0.0936724	newly	329.	347.43	{293., 406.}	11.6201	0.0480957	Reff	1.46491	2.52528	{2.31369, 2.91108}	72.3848	ar	93815.	89489.5	{86635., 93566.}	4.61201
39	R0	{1.90004, 12.3313, 1.}	{2.40716, 10.2286, 0.15}	{{1.74592, 5.58477, 0.}, {2.86001, 13.5542, 1.}}	{29.9373, 21.9132, 89.}	inf	121.	135.684	{78.8641, 190.051}	25.8646	0.150875	newly	167.	157.7	{112., 219.}	22.479	0.105489	Reff	1.89271	2.40153	{1.74269, 2.85277}	30.0926	ar	97413.	85737.5	{71437., 92945.}	11.9856
40	R0	{2.64218, 18.5237, 2.}	{2.2044, 5.2671, 0.14}	{{1.39162, 1.89883, 0.}, {2.8702, 8.24535, 1.}}	{20.6157, 71.5656, 93.}	inf	208.	115.064	{35.1145, 190.858}	58.7738	0.461608	newly	187.	451.43	{171., 790.}	148.829	0.330453	Reff	2.62693	2.19969	{1.39006, 2.86501}	20.5436	ar	99474.	79056.3	{50781., 93201.}	20.5257
41	R0	{1.6945, 7.17996, 0.1}	{1.99018, 11.5264, 0.74}	{{1.13726, 5.71232, 0.}, {2.77394, 17.4454, 2.}}	{31.6252, 69.5633, 726.}	inf	3.	4.25701	{1.31453, 8.25634}	47.9175	0.267137	newly	9.	5.7	{1., 9.}	80.	0.631004	Reff	1.69433	1.99004	{1.13723, 2.77347}	31.6264	ar	96332.	55737.2	{4., 92181.}	42.1405
42	R0	{1.84559, 18.0074, 1.}	{2.04217, 11.1615, 0.81}	{{1.67405, 7.28757, 0.}, {2.64334, 17.2462, 1.}}	{16.2942, 38.0171, 21.}	inf	1046.	958.194	{599.712, 1582.03}	39.6779	0.248561	newly	870.	821.53	{718., 921.}	8.58736	0.0404795	Reff	1.78061	2.00277	{1.64866, 2.57529}	17.706	ar	97358.	78037.5	{68169., 90849.}	19.8448
43	R0	{2.24965, 17.0361, 2.}	{2.70683, 11.8577, 0.67}	{{2.22376, 8.81055, 0.}, {2.9884, 13.7076, 1.}}	{21.8139, 30.3966, 66.5}	inf	172.	240.67	{169.679, 303.499}	17.1876	0.0797267	newly	161.	243.18	{176., 311.}	51.441	0.171923	Reff	2.23721	2.69645	{2.21683, 2.97684}	21.9723	ar	98770.	90962.2	{85205., 94030.}	7.90507
44	R0	{2.54324, 15.7967, 0.1}	{2.40159, 11.9659, 0.28}	{{1.93419, 8.56054, 0.}, {2.93371, 17.1903, 1.}}	{9.92719, 26.8347, 328.}	inf	68.	74.3662	{43.0076, 105.753}	36.9832	0.230951	newly	107.	68.	{41., 95.}	36.5981	0.220598	Reff	2.53714	2.39842	{1.93227, 2.92813}	9.86382	ar	99354.	86556.4	{77853., 93496.}	12.8808
45	R0	{2.54943, 14.8032, 0.1}	{2.55804, 8.68824, 0.13}	{{1.94705, 4.66877, 0.}, {2.93906, 10.6768, 1.}}	{15.1847, 41.3084, 206.}	inf	40.	38.2565	{10.2512, 59.5304}	37.3046	0.257896	newly	50.	71.8	{16., 105.}	74.48	0.248636	Reff	2.54637	2.55645	{1.94549, 2.93811}	15.2102	ar	99380.	86596.1	{76662., 93714.}	12.8636
46	R0	{2.37755, 12.6461, 2.}	{1.90826, 14.2916, 0.32}	{{1.16966, 8.89248, 0.}, {2.66225, 19.4013, 1.}}	{25.2439, 30.5482, 84.}	inf	8.	2.98484	{1.34642, 5.03524}	81.3448	0.791786	newly	26.	2.28	{0., 5.}	91.2308	1.31351	Reff	2.37696	1.90818	{1.16963, 2.66216}	25.2362	ar	99096.	55830.1	{4., 91203.}	43.6606
47	R0	{1.69279, 11.6636, 1.}	{2.3291, 15.9534, 3.23}	{{1.99822, 12.2033, 3.}, {2.7811, 19.6712, 4.}}	{37.5895, 37.5082, 223.}	inf	138.	245.931	{181.161, 347.108}	27.8235	0.100325	newly	167.	144.36	{103., 178.}	17.5808	0.0891878	Reff	1.68417	2.31926	{1.99115, 2.76713}	37.7089	ar	96214.	85735.9	{79625., 92366.}	10.8905
48	R0	{1.82402, 10.0038, 1.}	{2.42177, 8.99497, 0.17}	{{1.74592, 5.58477, 0.}, {2.9194, 11.0527, 1.}}	{34.941, 18.923, 85.}	inf	25.	35.7697	{14.4681, 55.8863}	36.9892	0.169645	newly	39.	52.29	{21., 83.}	56.1282	0.204095	Reff	1.82252	2.42028	{1.74524, 2.91721}	34.9595	ar	97076.	85928.7	{71188., 93485.}	11.4831
49	R0	{1.6424, 11.1667, 2.}	{1.64344, 5.11958, 0.2}	{{1.44762, 3.83841, 0.}, {1.74592, 5.83416, 0.}}	{12.1495, 55.6923, 93.}	inf	267.	186.024	{91.7839, 275.72}	55.0178	0.381324	newly	380.	399.34	{245., 561.}	25.5421	0.112533	Reff	1.62686	1.63543	{1.44189, 1.73765}	12.094	ar	95546.	63627.7	{54596., 71558.}	33.4062
50	R0	{2.05638, 14.3048, 1.}	{1.67095, 4.98132, 0.49}	{{1.24751, 2.19254, 0.}, {2.79531, 12.1249, 2.}}	{33.2026, 65.1773, 101.}	inf	167.	85.0427	{16.6409, 207.07}	63.0171	0.581178	newly	176.	212.11	{67., 308.}	53.892	0.213803	Reff	2.0461	1.66728	{1.24505, 2.78587}	33.0889	ar	98194.	54453.3	{15191., 92436.}	44.5452
